# AAV-Mediated angiotensin 1-7 overexpression inhibits tumor growth of lung cancer *in vitro* and *in vivo*

**DOI:** 10.18632/oncotarget.13396

**Published:** 2016-11-16

**Authors:** Xinglu Chen, Sansan Chen, Nana Pei, Yingying Mao, Shengyao Wang, Renhe Yan, Na Bai, Andrew Li, Yanling Zhang, Hongyan Du, Baihong Chen, Colin Sumners, Jinlong Li, Hongwei Li

**Affiliations:** ^1^ School of Laboratory Medicine and Biotechnology, Southern Medical University, Guangzhou, Guangdong, China; ^2^ Department of Urology, The First Affiliated Hospital of Clinical Medicine of Guangdong Pharmaceutical University, Guangzhou, Guangdong, China; ^3^ Department of Clinical Pathology, The First Affiliated Hospital of Jinan University, Guangzhou, Guangdong, China; ^4^ Deparement of Nuclear Medicine, People's Hospital of Yuxi City, Yuxi, Yunnan, China; ^5^ Department of Biomedical Engineering, The Johns University School of Medicine, Baltimore, USA; ^6^ Departments of Physiology and Functional Genomics, University of Florida, Gainesville, Florida, USA

**Keywords:** Ang-(1-7), lung cancer, adeno-associated viral vector, DNA synthesis, proliferation

## Abstract

Ang-(1-7) inhibits lung cancer cell growth both *in vitro* and *in vivo*. However, the molecular mechanism of action is unclear and also the rapid degradation of Ang-(1-7) *in vivo* limits its clinical application. Here, we have demonstrated that Ang- (1-7) inhibits lung cancer cell growth by interrupting pre-replicative complex assembly and restrains epithelial-mesenchymal transition via Cdc6 inhibition. Furthermore, we constructed a mutant adeno-associated viral vector AAV8 (Y733F) that produced stable and high efficient Ang-(1-7) expression in a xenograft tumor model. The results show that AAV8-mediated Ang-(1-7) over-expression can remarkably suppress tumor growth *in vivo* by down-regulating Cdc6 and anti-angiogenesis. Ang-(1-7) over-expression via the AAV8 method may be a promising strategy for lung cancer treatment.

## INTRODUCTION

Non-small cell lung cancer (NSCLC) accounts for over 80% of all lung cancers, and remains the most common cause of cancer-related death [1, 2]. Chemotherapy has been traditionally considered as one of the standard treatment options for advanced NSCLC, but the toxic side-effects and chemo-resistance hinder its clinical application [1]. Gene therapy has become a promising strategy for lung cancer therapies.

Ang-(1-7), an endogenous heptapeptide hormone of the renin-angiotensin system, mediates biological responses by activating Mas, a unique G protein-coupled receptor. Studies on Ang-(1-7) and Mas have mainly focused on hypertension and other cardiovascular diseases [3]. Interestingly, mounting evidence has shown that Ang-(1-7) exerts potent antitumor effects mainly through inhibitory effects on DNA synthesis, migration and invasion, and also antiangiogenic actions in a variety of tumors [4, 5]. Phase I/II dose escalation studies have assessed the safety and activity of Ang-(1-7) following chemotherapy in patients with breast cancer [6, 7]. However, the mechanisms underlying impairment of tumor proliferation are still unclear. Besides, the efficacy of this peptide *in vivo* is severely hampered due to rapid degradation by peptidases [7].

Here, we confirmed the inhibitory effect of Ang-(1-7) on proliferation and migration of NSCLC cells and demonstrated that the mechanism may be associated with suppressing pre-replicative complex (pre-RC) assembly and epithelial-mesenchymal transition (EMT). Moreover, we constructed a recombinant Adeno-associated virus (rAAV) vector to overexpress Ang-(1-7) continuously and stably in a murine xenotransplantation model of a human non-small cell lung cancer cell line (Spc-A1). The results indicate that overexpression of Ang-(1-7) inhibits tumor growth by decreasing Cdc6 and reduces angiogenesis by down-regulating VEGF.

## RESULTS

### Ang-(1-7) inhibits lung cancer cell proliferation by interrupting pre-RC assembly

**Figure 1:**
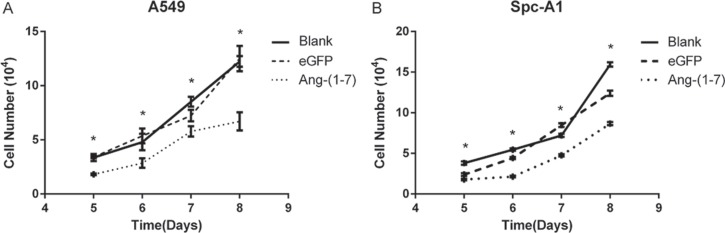
Ang-(1-7) inhibited growth of lung cancer cells A549 (**A**) or Spc-A1 (**B**) cells were infected with LV- Ang-(1-7) or LV-eGFP. Effect of Ang-(1-7) on cell proliferation was counted from day 5 after cells were plated. *P < 0.01 versus the control groups (LV-eGFP– and mock-transduced cells).

To verify the inhibitory effect of Ang-(1-7) on lung cancer cells, A549 and Spc-A1 cells were infected with lentiviral vector LV-Ang-(1-7) or LV-eGFP at 200 vg/cell respectively. Transfected cells were plated into a 24-well cluster dish and then were counted on day 5, 6, 7 and 8 to determine the cell growth. The data demonstrated that cell growth was inhibited in the LV-Ang-(1-7) transduced cells compared to the LV-eGFP or mock transduced cells (Figure 1).

**Figure 2:**
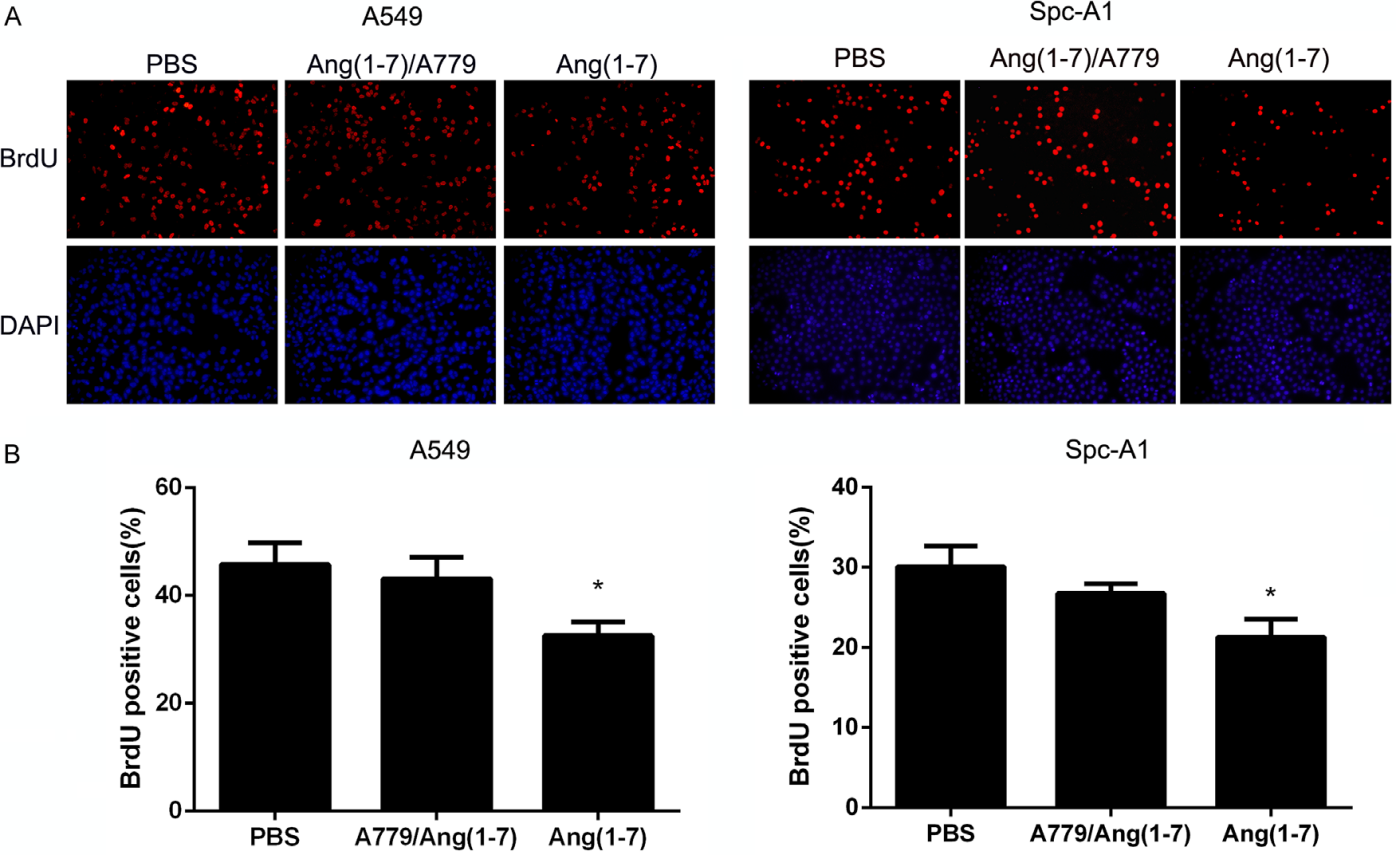
Ang-(1-7) inhibited DNA synthesis in lung cancer cells (**A**) A549 and Spc-A1 cells were treated with PBS or 500 nmol/L Ang-(1-7) with or without 1 μmol/L A779 for 24 h, then incubated with BrdU for 2 h. (**B**) The BrdU-positive cells were identified by immunofluorescence staining and observed under a fluorescence microscope. *P < 0.05.

A BrdU incorporation assay was used to determine whether Ang-(1-7) inhibited DNA replication of lung cancer cell lines. A549 and Spc-A1 lung cancer cells were treated with 500 nM Ang-(1-7) for 24 h. Next, 10 μM BrdU was added and incubated with cells for 2 h, followed by immunofluorescence detection (Figure 2A). As shown in the summary data in Figure 2B, the number of BrdU positive cells in the Ang-(1-7) group was significantly lower than in control groups, indicating that Ang-(1-7) reduced DNA synthesis. It has been demonstrated that Ang-(1-7) exerts its biological effects by binding to the Mas receptor [8, 9]. A779, a Mas receptor antagonist, attenuated the inhibitory effect of Ang-(1-7) on DNA synthesis.

**Figure 3:**
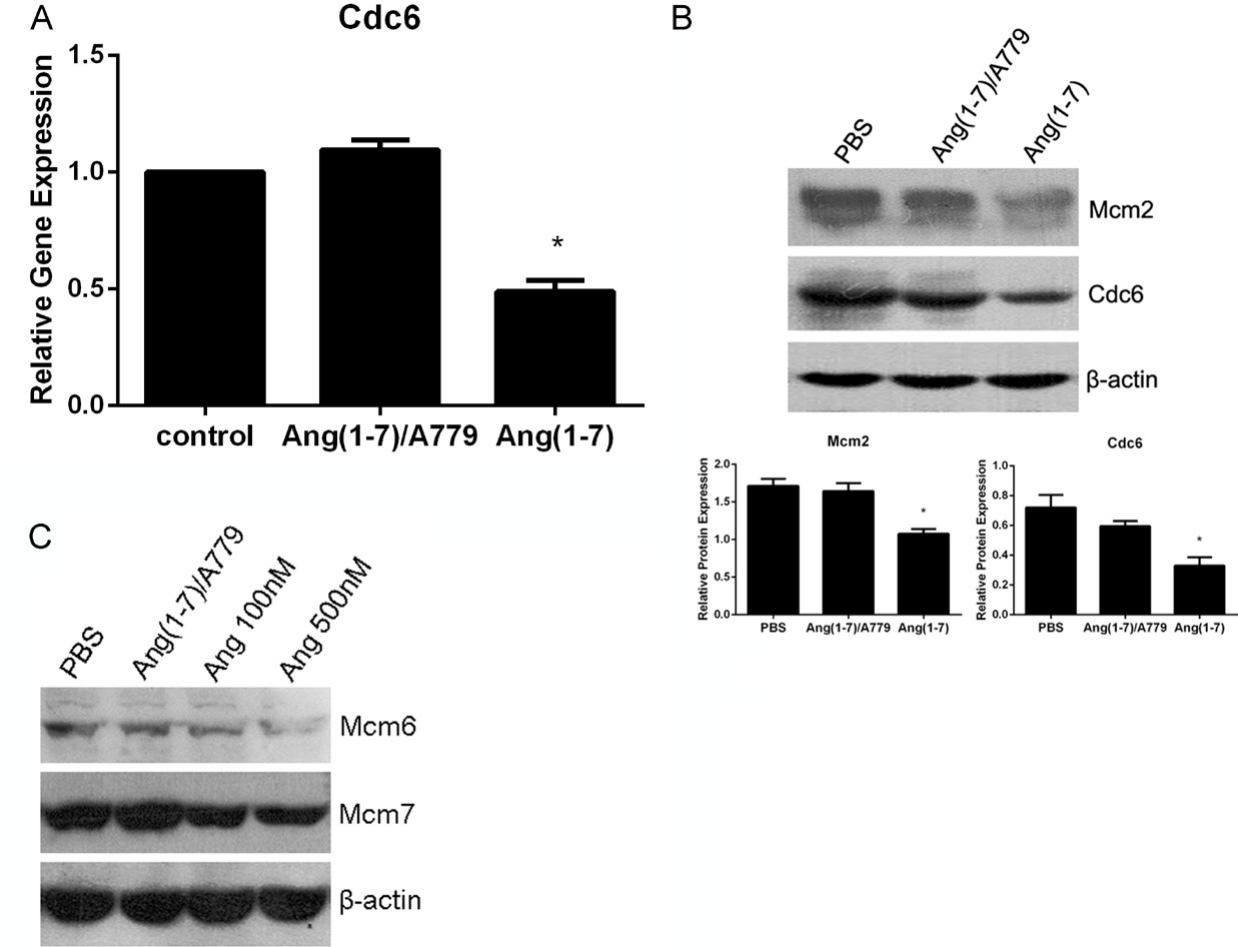
Ang-(1-7) downregulates Cdc6 mRNA and protein levels in Spc-A1 cells Spc-A1 cells were treated with PBS or 500 nM Ang-(1-7), with or without 1 μM A779 for 24 h. Cells were collected and subjected to real-time RT-PCR and Western blot analyses (data are representative of three different experiments). (**A**) Expression level of Cdc6 in Spc-A1 cells after treatment with Ang-(1-7) compared to control groups. *P < 0.01. (**B**) Expression of Cdc6 and Mcm2 along with *β*-actin protein in Spc-A1 cells. Western blots are representative of three different experiments. *P < 0.01. (**C**) Expression of Mcm6 and Mcm7 protein in Spc-A1 cells treated with PBS, 1 μM A779 with 500 nM Ang-(1-7), 100 nM or 500 nM Ang-(1-7).

Cdc6 plays a crucial role in the assembly of pre-replication complexes (pre-RCs) by linking the origin recognition complex (Orc) with minichromosome maintenance (Mcm) proteins to form pre-RCs at the sites of DNA replication. RT-PCR results from lung cancer Spc-A1 cells showed that Cdc6 mRNA level was significantly decreased by Ang-(1-7) (Figure 3A). Accordingly, Western blot analysis demonstrated that Ang-(1-7) decreased Cdc6 protein level in Spc-A1 cells (Figure 3B). In addition, Mcm2, a component of pre-RCs, was also down-regulated following Ang-(1-7) treatment (Figure 3B). Therefore, the data suggest that Ang-(1-7) may inhibit pre-RCs assembly, and point to a crucial role of Cdc6 and Mcms in this process. Down-regulation of Cdc6 mRNA and Cdc6 and Mcm2 proteins by Ang- (1-7) were abolished by the Ang-(1-7) antagonist, A779 (Figure 3A, 3B).

To further investigate the role of Ang-(1-7) in pre-RCs assembly, we analyzed the expression of pre-RCs in the chromatin-binding fraction. As shown in Figure 3C, Mcm6, and Mcm7 loaded on chromatin were significantly decreased after Ang-(1-7) treatment, effects that were abolished by A779. These results suggest that Ang-(1-7) can disrupt pre-RCs assembly and subsequently inhibit DNA synthesis.

### Ang-(1-7) suppresses EMT in NSCLC

**Figure 4:**
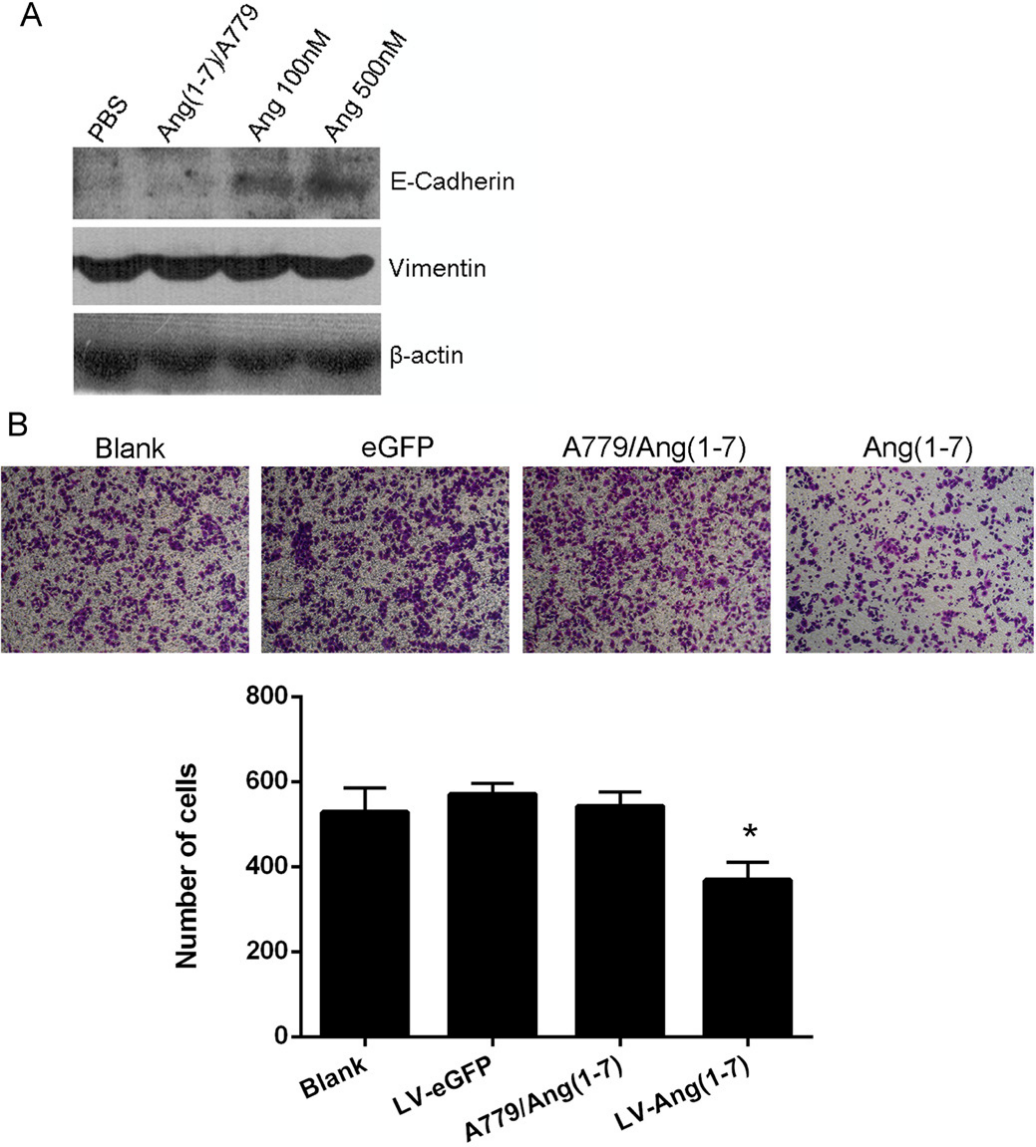
Effects of Ang-(1-7) on Spc-A1 cell EMT (**A**) Western blot analysis of E-Cadherin and Vimentin protein expression in Spc-A1 cells after treatment with PBS, 1μM A779 with 500 nM Ang-(1-7), 100 nM or 500 nM Ang-(1-7). Western blots are representative of three different experiments. (**B**) *In vitro* transwell migration assay. Non-transduced or Lenti-Ang-(1-7) transduced Spc-A1 cells were resuspended in serum free DMEM with or without 1.0 μmol/L the Ang- (1-7) receptor antagonist, A-779, and placed in the top portion of a transwell chamber. Migration ability of Spc-A1 cell was quantified by counting numbers of cells that adhere to the outside surface of the membranes. Five fields per well were counted. Values are expressed as mean ± SD of three independent experiments. *P < 0.05, vs.controls.

It has been demonstrated that Cdc6 promotes epithelial to mesenchymal transition (EMT) [10]. Thus, we hypothesized that Ang-(1-7) may inhibit EMT in NSCLC due to its role in lowering Cdc6 expression and analyzed the protein levels of several epithelial and mesenchymal markers. Accordingly, the protein level of the epithelial marker E-cadherin was elevated by Ang-(1-7) treatment, while the levels of vimentin, a mesenchymal marker, were not altered (Figure 4A).

Tumor cells undergoing EMT tend to lose cell-cell adhesiveness [11]. Since Ang-(1-7) can restrain EMT in NSCLC cells, it may exert inhibitory effects on migration. Consistent with our speculation, the migration ability of Spc-A1 cells declined after Ang-(1-7) treatment, an effect that was blocked by the Mas antagonist, A-779 (Figure 4B).

### Ang-(1-7) inhibits p38 MAPK signaling pathway

**Figure 5:**
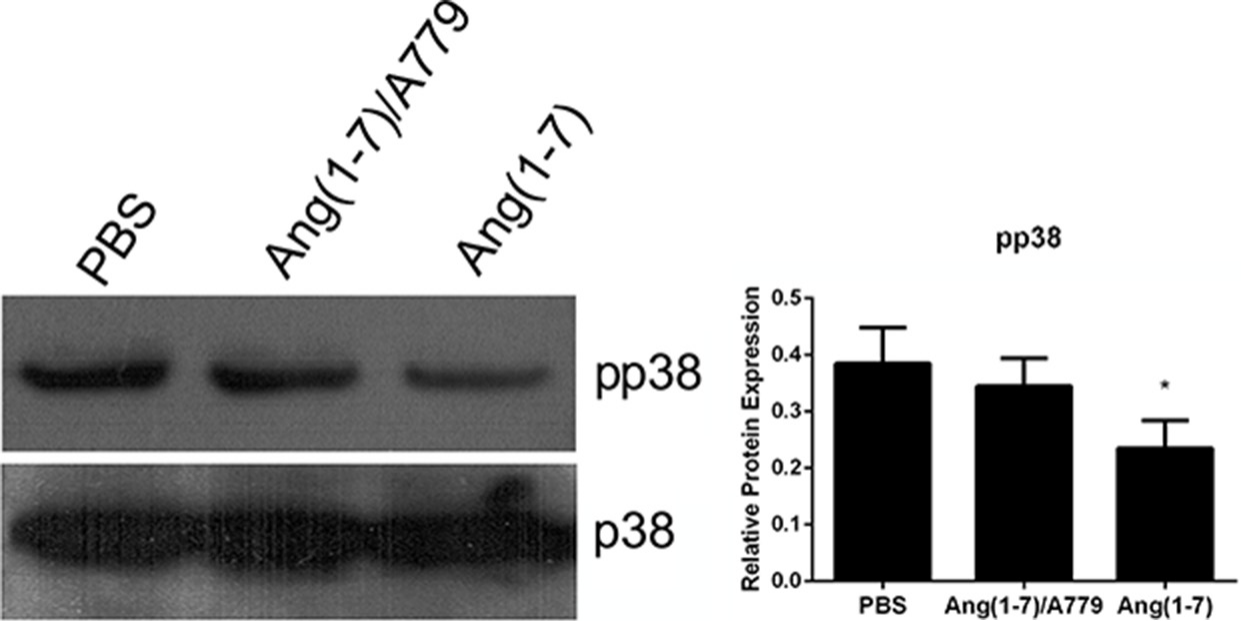
Effects of Ang-(1-7) on p38 MAPK in Spc-A1 cells Spc-A1 cells were treated with 500 nM Ang-(1-7) with or without 1 μM A779. After 3 days of incubation, cells were collected and subjected Western blot analyses of total p38 and pp38. Western blots are representative of three different experiments. *P < 0.01.

The p38 MAPK signaling pathway plays a vital role in cell proliferation and migration. The p38 phosphorylation was decreased by Ang-(1-7) treatment as compared with the saline group, an effect that was reversed by A779 (Figure 5).

### Ang-(1-7) inhibits lung tumor growth via anti-proliferative and anti-angiogenic effects

**Figure 6:**
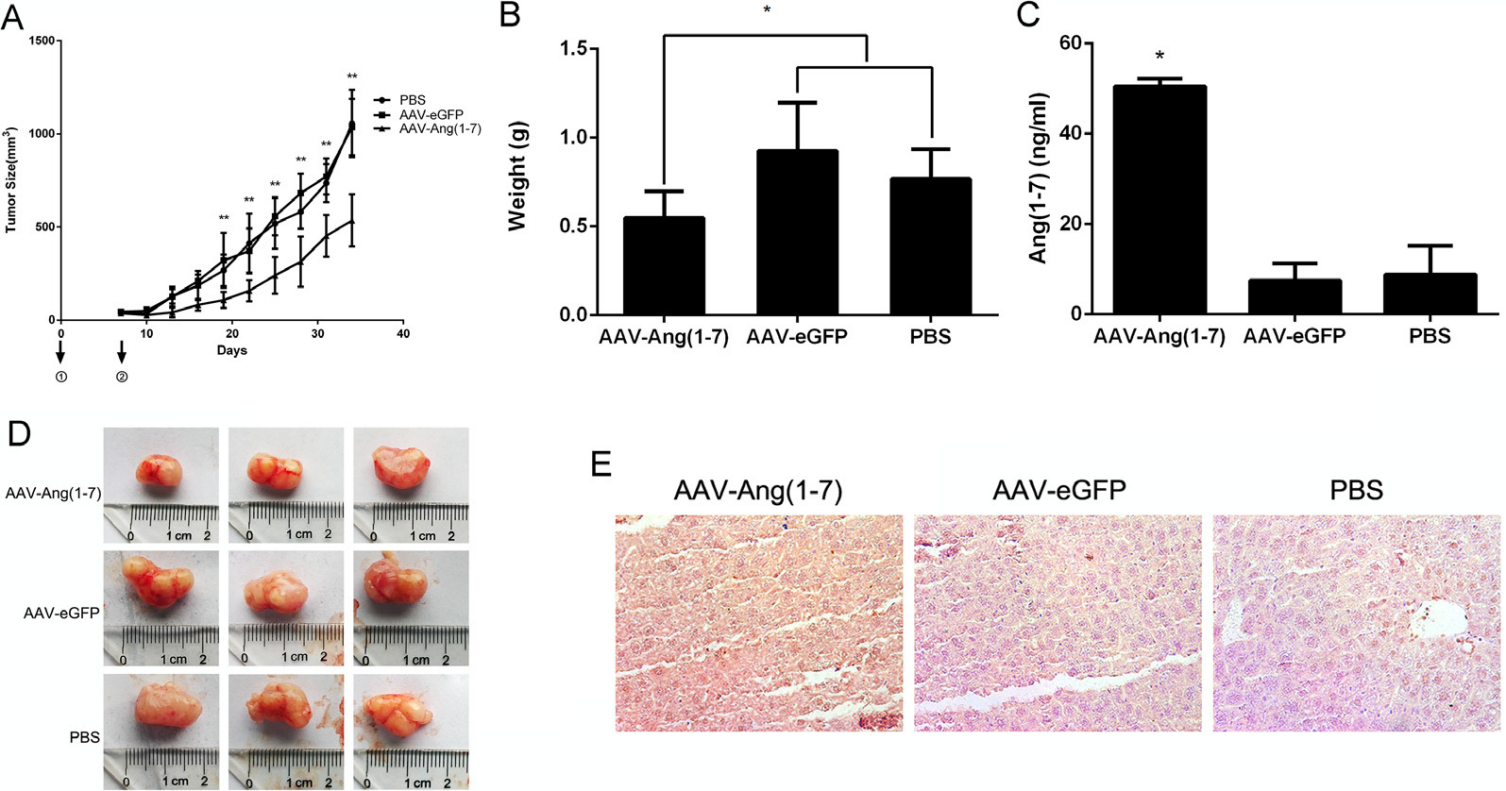
Ang-(1-7) inhibits lung tumor growth (**A**) Growth curve of lung tumor xenografts volumes. Volume = (D × d^2^)/2, where D is the longest diameter and d is the shortest diameter; *P < 0.01; *n* = 6 (**B**) Tumors from mice treated with AAV8 (Y733F)-CBA-Ang-(1-7), AAV8 (Y733F)-CBA-eGFP, or PBS were weighed at the time of sacrifice; *P < 0.01; *n* = 6. Each data point represents the mean ± SD of 6 mice. (**C**) Levels of Ang-(1-7) in the sera of controls (AAV-eGFP and PBS-treated mice) and AAV8 (Y733F)-CBA-Ang- (1-7)-treated mice, which were determined by ELISA. *P < 0.01 compared with the control groups. (**D**) Size of human lung tumor xenografts from mice injected with AAV8 (Y733F)-CBA-Ang-(1-7), AAV8 (Y733F)-CBA-eGFP, or PBS. (**E**) Sections of livers from treated mice were stained with mouse IgG to indirectly determine Ang-(1-7) overexpression. Representative photomicrographs are shown.

The short elimination half time of Ang-(1-7) *in vivo* limits its clinical use [7]. Hence, we constructed an AAV vector, AAV-Ang-(1-7), to express the Ang-(1-7) consistently and stably. When xenograft tumor volumes were ~50 mm^3^, mice were treated with AAV-Ang-(1-7), AAV-eGFP (1 × 10^11^ vg/mice/100 μl) or PBS by a single intravenous injection into the tail. Tumor volumes were measured every 3-days for a period of 34 days. The tumor volume in the AAV-Ang-(1-7)-treated group was significantly smaller than in AAV-eGFP and PBS groups from 19 days after injection, while there were no differences between the AAV-eGFP and PBS groups (Figure 6A). At the end of the study, the tumor weight and size in AAV-Ang-(1-7) group was markedly reduced compared with the control groups (Figure 6B, 6D).

To verify that AAV-Ang-(1-7) produced steady Ang-(1-7) expression, the Ang-(1-7) secreted into the serum was quantified by ELISA. The results showed that Ang-(1-7) was significantly increased in AAV-Ang- (1-7)-treated mice compared with the AAV-eGFP or PBS groups (Figure 6C). Because of AAV mediated Ang-(1-7) expression targeting liver, fusion protein containing mouse IgG Fc region could be detected via immunostaining using horseradish peroxidase-conjugated anti-mouse IgG (Figure 6E).

**Figure 7:**
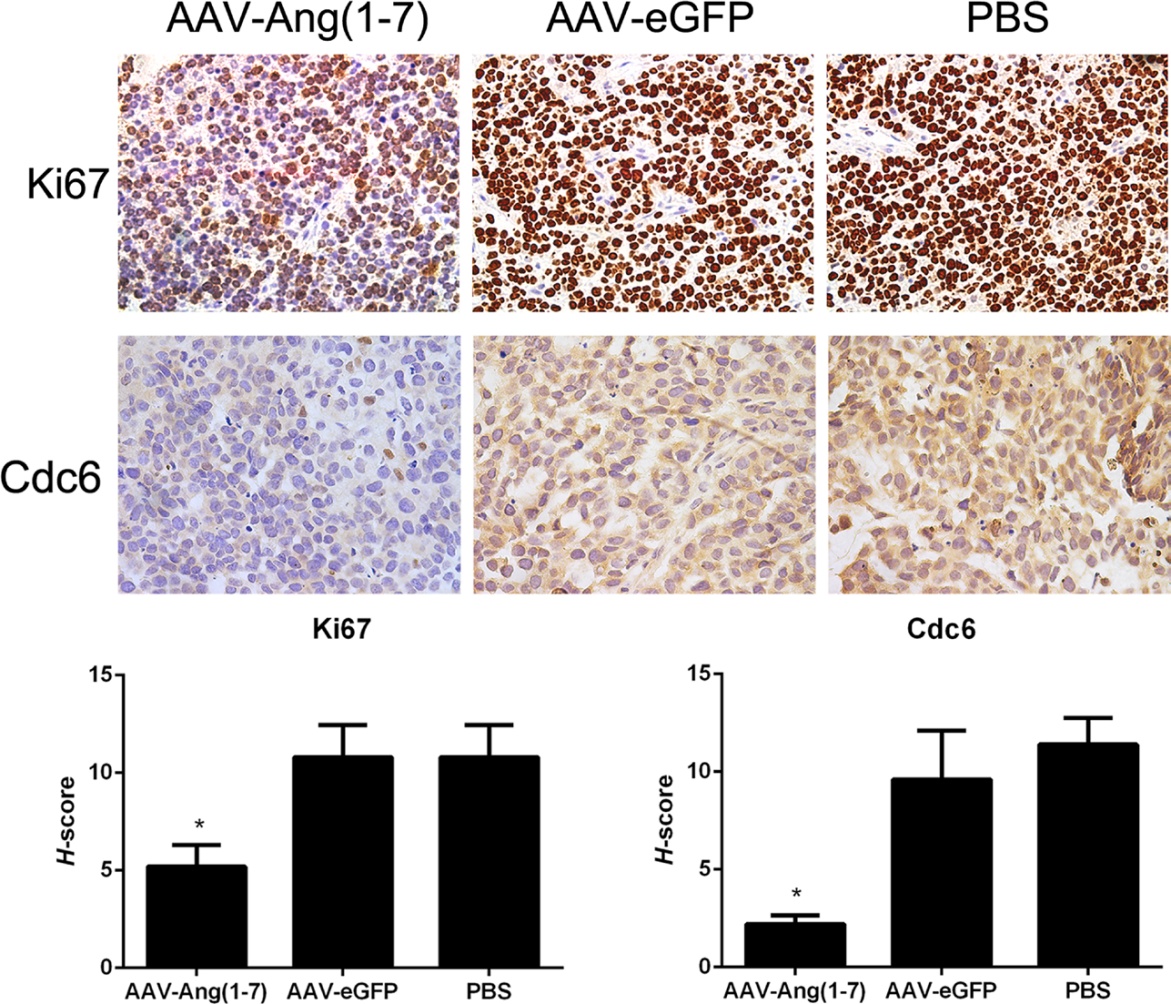
Ang-(1-7) reduces Spc-A1 xenograft tumor cell proliferation Sections of transplanted tumors infused with AAV8 (Y733F)-CBA-Ang-(1-7), AAV8 (Y733F)-CBA-eGFP, or PBS were stained with Ki67 and Cdc6 antibodies. Representative photomicrographs are shown at the top(magnification, × 400); The histochemical score (*H*-score) as the final score was achieved by the intensity multiplied by the percentage of positive cells; bars, SD. *P <0.01 vs. the control groups (AAV-eGFP and PBS-treated mice).

Tumor paraffin sections were stained with Ki67 antibodies as shown in Figure 7A. Ki67 is a nuclear protein presented in the active phases of the cell cycle (G1, S, G2, and M), but is not expressed in quiescent cells. Tumor sections from Ang-(1-7)-treated animals stained with anti-Ki67 antibodies showed a significantly less percentage of stained cells compared with the two control groups, suggesting that Ang-(1-7) reduces Spc-A1 xenograft tumor cell proliferation.

High levels of Cdc6 have been reported in around 50% of non-small cell lung carcinomas [12], brain cancer [13] and a subset of mantle cell lymphomas [14], which suggests that Cdc6 has oncogenic properties. Tumor sections stained with anti-Cdc6 showed significantly decreased Cdc6 immunostaining following treatment with Ang-(1-7) (Figure 7B).

### Ang-(1-7) reduces vessel density in Spc-A1 xenograft tumors

**Figure 8:**
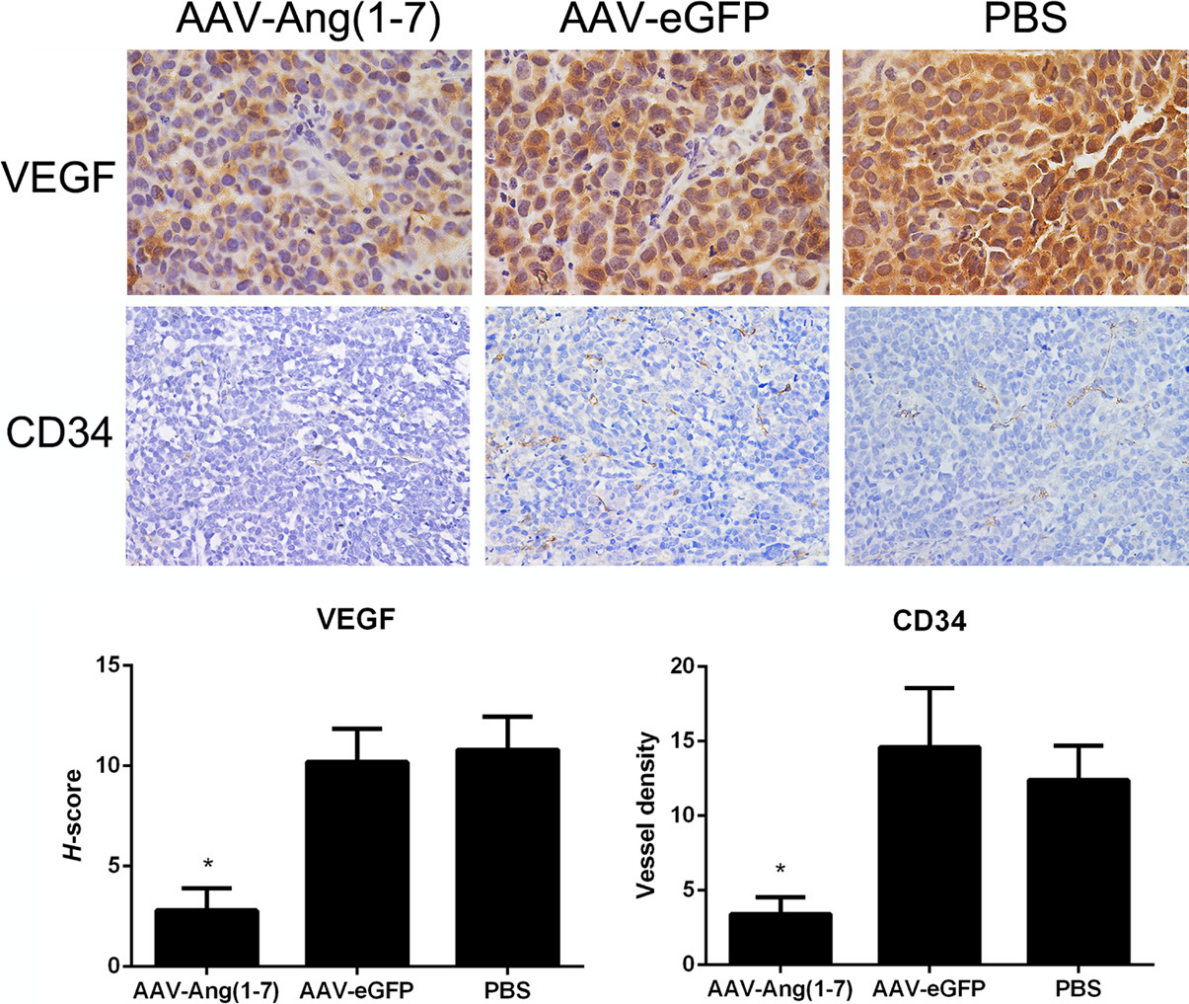
Effect of Ang-(1-7) on angiogenic factors and vessel density in lung tumor xenografts Ang-(1-7) decreases VEGF expression in human lung cancer xenografts. VEGF expression in tumor sections from AAV8 (Y733F)-CBA-Ang-(1-7), AAV8 (Y733F)-CBA-eGFP or PBS-treated human lung cancer xenografts were determined through VEGF immunostaining (magnification ×400). Blood vessels in tumor sections from lung cancer xenografts were identified by CD34 immunoreactivity and vessel morphology, and quantified as the average of 5 fields selected per tumor (magnification ×200). Columns are means from 5 separate experiments; bars, SD. *P < 0.01 vs. the control groups (AAV-eGFP and PBS-treated mice).

In order to identify whether AAV-Ang-(1-7) inhibits angiogenesis within lung tumor xenografts, blood vessels were detected by immunohistochemical staining using VEGF and CD34 antibodies. Tumor sections stained with antibodies against vascular endothelial growth factor (VEGF), exhibited significantly less stained cells in the Ang-(1-7)-treated group compared with the two control groups, suggesting that Ang-(1-7) reduces Spc-A1 xenograft tumor cell angiogenesis (Figure 8A). Vessels were identified by positive immunoreactivity to CD34 in combination with vessel morphology. A marked reduction in immunoreactive CD34-stained vessels was observed in tumor tissue sections from mice treated with AAV-Ang-(1-7) (3.5 ± 1.5 vessels/field) when compared with mice treated with AAV-eGFP (15 ± 5 vessels/field) or PBS (13 ± 3 vessels/field; Figure 8B), suggesting that Ang-(1-7) significantly attenuates tumor vascularization in xenograft lung tumors.

## DISCUSSION

In this study we investigated the effects of Ang-(1–7) on the growth of lung cancer *in vitro* and *in vivo*, by using lentiviral or AAV vectors expressing fusion proteins which release the heptapeptide. Ang-(1-7) has been shown to inhibit the growth of lung cancer cells through a MAP kinase-dependent mechanism, and attenuated the DNA synthesis in SK-LU-1 cells, effects that were blocked by the Mas receptor antagonist A779 [4]. In the current study, we observed the significant Ang-(1-7)-induced inhibition of the growth of and DNA synthesis in Spc-A1 and A549 (Figures 1 and 2). Cell division cycle 6 (CDC6) is an essential regulator of DNA replication in eukaryotic cells. Its best-characterized function is the assembly of pre-replicative complexes at origins of replication during the G1 phase of the cell division cycle [15]. Prior to DNA replication, pre-replication complexes (pre-RCs) must be assembled at the replication sites in cells in the G1 phase [16]. Cdc6 plays a crucial role in the assembly of pre-RCs by linking the origin recognition complex (Orc) with minichromosome maintenance (Mcm) proteins to form pre-RCs at the sites of DNA replication [17]. Our results revealed that the Ang-(1-7) peptide may inhibit DNA synthesis by decreasing the pre-RCs protein levels, including CDC6 and Mcms (Figure 3).

High levels of CDC6 have been recently reported in around 50% of NSCLC [12], brain cancer [13] and a subset of mantle cell lymphomas [14], which suggests that CDC6 has oncogenic properties. Our study demonstrated that CDC6 was significantly decreased in lung cancer cells treated with Ang-(1-7), both *in vitro* or *in vivo* (Figure 7).

It has been reported that overexpression of CDC6 induced EMT [10], and that activation of p38 triggers EMT and cell migration [18]. Our results indicated that Ang-(1-7) inhibited Spc-A1 cells from undergoing EMT and cell migration (Figure 4), and also inhibited phospho-activation of p38 MAPK which mediated cell migration [19–22] (Figure 5) was decreased as well. The down-regulation of p38 MAPK, ERK1/2, and Akt decreased the protein expression of CDC6 and the activation of the p38 MAPK, and ERK1/2, and Akt pathways are required for the protein stabilization of CDC6 [23]. Based upon this, we suggest that EMT can be a new mechanism in the further research of Ang-(1-7).

Interestingly it has been demonstrated that Ang- (1-7) enhanced the migratory and invasive abilities of renal cell carcinoma 786-O and Caki-1 cells in wound-healing, transwell migration and transwell invasion assays [24], effects that are opposite to those in our experiments on lung cancer cell migration (Figure 2). These inconsistent results suggest that the actions of Ang-(1-7) are possibly tumor-specific and highly dependent on its target in different cancer cells [25].

Using mouse models, many studies have shown that Ang-(1-7) exerts effects on tumor growth. Namely, it elicits markedly reduced tumor volume and wet weight of human A549 lung tumors grown in the flank [5, 26]; of orthotopic human breast tumors positive for the estrogen receptor (BT-474 or ZR-75-1) and over-expressed HER2 (BT-474) in the mammary fat pad [27]; of human LNCaP prostate cancer xenografts [28] as compared to tumors from control animals [3]. All of these studies treated the mice with Ang-(1-7) by inserting minipumps to produce continuous infusion, because of the rapid degradation of the peptide. Our previous study demonstrated that administration of AAV8-(Y733F)-CBA-Ang-(1-7) significantly attenuated the growth of human nasopharyngeal carcinoma xenograft tumors, suggesting that AAV-based gene therapy is potentially effective and useful for nasopharyngeal cancer treatment [25]. In the present study, we used recombinant adeno-associated virus AAV8-(Y733F)-CBA-Ang-(1-7) to treat nude mice with lung cancer xenografts. The results demonstrated that the growth of Spc-A1 lung cancer cells xenografts were inhibited by AAV8-Ang-(1-7) *in vivo* (Figure 6A), through the inhibition of cell proliferation and angiogenesis (Figures 7, 8). After a single injection through the tail vein, a high level of Ang-(1-7) was express stably for a month, as detected by ELISA (Figure 6C). Moreover, AAV vectors have been utilized in many clinical trials, which have proven their safety and their therapeutic efficacy in monogenic diseases and also cancers.

In conclusion, our research has demonstrated that Ang-(1-7) inhibited the growth of lung cancer cells, an effect that is probably mediated by the inhibition of DNA synthesis. AAV8-(Y733F)-CBA-Ang-(1-7) significantly attenuated the growth and angiogenesis of lung cancer cells xenograft, suggesting that AAV-based gene therapy can be a useful and efficient approach for treating lung cancer.

## MATERIALS AND METHODS

### Cell culture

Human lung cancer cell lines (A549 and Spc-A1) were obtained from the American Tissue Culture Collection and were cultured in Dulbecco's modified Eagle's medium (DMEM; Corning) supplemented with 10% FBS under 5.0% CO_2_. Sera and media were purchased from Invitrogen and American Type Culture Collection. HEK 293T cells were cultured in Dulbecco's modified Eagle's medium (DMEM; Corning).

### Viral vector construction and preparation

The lentiviral vector LV-Ang-(1-7) construct used to produce Ang-(1-7) was designed, prepared, and titrated as previously described [7, 29, 30]. Both IgG2b and Ang- (1-7) from the fusion protein can be detected intracellularly. The enhanced GFP (eGFP) lentiviral vector LV-eGFP was used as a control. Two adeno-associated viral vectors (AAV), AAV2-chicken β-actin promoter (CBA)-Ang-(1-7) and AAV2-CBA-eGFP, were constructed as detailed previously [31]. The vector plasmid was packaged in AAV serotype 8 containing a Y733F mutation by transfection of HEK 293T cells according to previously published methods [32, 33]. Vector doses were expressed as genome copies.

### Quantification of cell proliferation

For the cell counting assay, cells were infected with LV-Ang-(1-7) to stably overexpress Ang-(1-7). After 72 hours of transduction, the cells were plated in 24 well plates at 1.0 × 10^4^ per well. Cells were harvested on days 1, 2, 3, and 4 from triplicate wells and counted using a hemocytometer, to quantify the cell proliferation.

### *In vitro* migration assay

Cell migration assays were performed using 24-well Transwell plates (8-μm pore size; Corning). Confluent monolayers of Spc-A1 cells transduced with LV-eGFP and LV-Ang-(1-7) with or without A779 were cultured in serum free media in the upper chambers (5 × 10^4^ cells per chamber). The lower chambers were filled with medium DMEM, 10% FBS. Transwell plates were then incubated at 37°C for 24 hours. Cells migrated to the outer side of the upper chamber were fixed with methanol for 30 min, PBS washed three times, and then stained with Wright Giemsa staining. The number of migrated cells was assessed by counting cells from 5 random fields per well. Each experiment was performed in duplicate and repeated three times.

### BrdU incorporation assay

The proliferation of A549 and Spc-A1 cells were determined via 5-bromo-2′deoxyuridine-5′monophosphate (BrdU) incorporation assay. A549 and Spc-A1 cells treated with PBS or 500 nM Ang-(1-7) were seeded into 6-well plates (3 × 10^5^ cells/well) with or without the A-779 (1.0 μmol/L) (Bachem) for 24 h. Two hours prior to fixing the cells, 20 μM BrdU (Sigma chemicals) were added to the cultures. Next, the cells were permeabilized with 0.25% Triton X-100 and 3 M hydrochloric acid. The cells were blocked in goat serum albumin. Then, immunofluorescence was performed as previously described [34] by incubation with an anti-BrdU antibody (1:1000, Cell Signaling Technology, USA) at 4°C overnight. The cells were then washed with cold PBS and incubated with Goat anti-Mouse IgG (H+L) Secondary Antibody, Cy3 conjugate (Invitrogen, USA) for 1 h at room temperature. Labeling indices were calculated as the number of positively stained cells divided by the number of total cells.

### RNA isolation, reverse transcription, and quantitative real-time RT-PCR

Total RNA was extracted using a HiPure Total RNA Kit (Magen) according to the manufacturer's instructions. Quantitative realtime RT-PCR was performed on an ABI 7500 real-time PCR system (Applied Biosystems) as described previously [35]. The samples were quantified by the comparative ΔΔCt method by using human GAPDH as the internal standard.

### Western blotting

Cell lysis and western blotting were performed as described previously [36]. Immunoreactive bands were visualized by enhanced chemiluminescence detection reagents and analyzed with Image J 1.62. For cell signaling study, 2 *×* 10^5^ cells were seeded into 6-well plates and allowed to settle for 24 h. Then the cells were starved in serum-free medium for 24 h before stimulation with Ang-(1-7) or Ang-(1-7)/A779.

### Tumor growth assay

Female BALB/c nude mice aged 4 to 5 weeks were purchased from the Institute of Comparative Medicine and Center of Laboratory Animals of the Southern Medical University (SMU). Animal handling and experimental procedures were approved by the Animal Experimental Ethics Committee of SMU. Athymic mice were subjected to s.c. injections of human Spc-A1 lung cancer cells (1.0 × 10^6^) in Matrigel (50:50) into the lower flank to induce tumor growth. After the tumors reached ~50 mm^3^, the mice were placed into three groups at random and the animals received tail vein injections of AAV8 (Y733F)-CBA-Ang-(1-7), AAV8 (Y733F)-CBA-eGFP (1 × 10^11^ vg/mice) or PBS. Each group contained 5 mice and the experiment was repeated 3 times. Tumor size was measured every 3 days. The mice were anesthetized on day 32 and euthanized by decapitation and tumors were dissected. Tumor volumes were calculated as follows: volume = (D × d^2^)/2, where D meant the longest diameter and d meant the shortest diameter. Both livers and tumors were fixed in 10% buffered formalin and used for histologic and immunohistochemical analysis. Sera were also collected for quantification of Ang-(1-7) by ELISA (Bachem).

### Immunohistochemistry

Tumors were fixed in formalin for 24 hours and incubated in 70% ethanol for 48 hours before embedding in paraffin. The embedded tumors were cut into 5-mm-thick sections and stained with H&E to determine morphology. Cell proliferation in the transplanted tumors was analyzed using antibodies to Ki67 (1:200; Abcam) and Cdc6 (1:300; Abcam) expression. Angiogenesis was determined by immunostaining with antibodies to VEGF (1:100; Wanleibio, China) and CD34 (1:200; Abcam). Visualization was achieved using the EnVision+ peroxidase system (Dako) according to the manufacturer's protocols. Ki67- immunoreactive cells were expressed as a percentage of the total cell number of examined fields. Blood vessels were visualized by the presence of CD34-immunostained endothelial cells and identified by their morphology, as vessels cut in cross-section with visible lumens or vessels cut longitudinally with tube-like morphology [37]. The vessel density was assessed by counting vessels from five random fields per tumor. Counts were done by an individual who was blinded as to the treatment.

### Evaluation of immunostaining scores

The intensity of staining was scored by applying a semiquantitative system, ranging from negative to strong as follows: 0 (negative), 1 (weak), 2 (moderate), and 3 (strong). The percentage of positive cells was categorized according to the positive tumor cells: < 25% is 1; 25% to 50% is 2; 51% to 75% is 3; and > 75% is 4. The histochemical score (*H*-score) as the final score was achieved by the intensity multiplied by the percentage of positive cells [38, 39].
